# Syphilitic Locolosis Alveolaris (Pyorrhœa Alveolaris)

**Published:** 1900-10

**Authors:** G. Lenox Curtis

**Affiliations:** New York City


### Abstracts and Translations.

### SYPHILITIC LOCOLOSIS ALVEOLARIS (PYORRHŒA ALVEOLARIS).[1]

BY G. LENOX CURTIS, M.D., NEW YORK CITY.

For more than twenty years I have given this subject close attention, not only with a view to its cure, but to ascertain the etiology of this disease, which is generally regarded as incurable.

Dr. Farrar says, "I believe locolosis alveolaris is a disease of the peridental membrane aggravated by calcareous deposits upon the teeth, which increase the inflammation so greatly that decalcification of the alveolar tissue results, and when this state exists the

---

[1] Read before the American Medical Association, Atlantic City, June 7, 1900.

advance of locolosis increases more rapidly until nature makes a serious effort to expel the tooth, and if successful the disease subsides and is lost from view. When all the teeth are lost locolosis ceases to be observed, showing that whatever the cause of the socket disease, it does not reappear elsewhere."

In an article, "Some Suggestions on the Treatment of Pyorrhœa Alveolaris," by myself, published in the *New York Medical Journal* of January 14, 1899, I gave my views in regard to its treatment. I propose now to give some of my views upon the causes of this old and destructive disease.

Up to about twelve years ago I had treated several hundred cases of what I then supposed to be pyorrhœa alveolaris. But in the light of later knowledge I am of the opinion that only recurring cases were worthy of that appellation. At that time I boldly resorted to all methods then in vogue, and freely referred my cases in consultation to those whom (from their writings) I believed to know most on the subject. Degeneracy, due to uric acid, and rheumatism were suggested, and as I found the rheumatic and gouty tendency in some patients having the disease, I inclined to accept them as a cause, if not the cause. But later, when the treatment did not effectually suppress the disease, I was satisfied there must be something else behind it all which should be learned. Reviewing the history of many of the most obstinate cases, I found that in several I could trace syphilitic association. Believing much information might be gained, I followed this trail, and have continued so doing from that time. It was difficult to secure sufficient data, however, by which I could prove scientifically that which I suspected, for, as we all know, syphilis has such an insidious undermining effect, and patients are generally so unwilling to admit facts, that the study has many discouraging aspects. It is, however, my opinion that this disease does not alone show itself in those persons who have contracted it, but may also be found in the mother as well as her offspring. It was in these cases that I found locolosis or pyorrhœa alveolaris to be so well defined that I felt encouragement. But obstacles arose which retarded my speedily reaching a definite conclusion. It was my hope at this time that I might gain something by turning these cases over to specialists in syphilis, gout, and rheumatism for treatment, but the varied results led me to suspect, and to be cautious in speech until I could get sufficient verified data to act more intelligently.

In many cases I found that treatment had not been continued sufficiently to eradicate the specific poison or the secondary effects thereof. In 1890 I had an opportunity to study blood, and then it was that I became convinced that the usual method of physiological study of this pabulum was inadequate. I now believe the blood carries with it the active principles of most, if not all, diseases. Then the generally accepted plan for the examination of the blood was through dry and stained specimens. Even to-day that plan is largely followed. Could any but the most tenacious germs stand the baking process which is claimed to be unavoidable? Not only are such specimens exposed to oxidizing influence of the atmosphere, but to heat which is of such a temperature that it is injurious to them. It might be said that only the survival of the fittest can furnish the possible opportunity of study, and then they can be recognized only after a course of staining that decorates them in "war paint," chiefs of their tribe. In Von Ziemssen's "Practice of Medicine," vol. iii., page 40, we find that Kircher in 1695 claimed disease to be due to living organisms, but it was not until 1772 that Lostorfor claimed to be able to distinguish, by microscopical examination of the blood, the presence of syphilis and other diseases.

In 1890 Watkins, after studying various methods of blood preparation, came to the conclusion, and published the fact, that there was only one method of scientifically examining the blood,—namely, doing it in its fresh state, and before any changes had taken place. He also found that by instantaneous photographing fresh blood, objects which would otherwise be overlooked would be revealed and permanently recorded, showing facts that the dry and stained specimens would fail to do.

In 1892 my attention was called to Dr. Watkins's method, and it so favorably impressed me that I have since devoted considerable time to it, and now I am so convinced that it is the only road to an accurate diagnosis of disease that I am still continuing the accumulation of data, with more or less satisfaction, having for my chief guide a sign in the mouth which I first observed many years ago, but the importance of which I then failed to appreciate. This sign, which I denominate "egg-skin eschar," I find upon the mucous membrane extending along the ramus and the buccal surface of the gums along the molars. Occasionally it is to be found upon the cheek, near Steno's duct and the angle of the mouth. But I

will not dwell upon this point, as I referred to it in the discussion of my article above alluded to.

In the early treatment of this disease, when I found this eschar present, as it was in many cases, I learned to associate it with some obstinate forms.

Five years ago I began sending patients to Dr. Robert L. Watkins, of New York City, for blood examination, with the view of ascertaining what existed. This I did without giving Dr. Watkins the history of the case. The examination of more than one hundred cases revealed strong evidences of syphilis, and in every instance when the egg-skin eschar was found the blood showed unmistakable proofs of the taint; in fact, every case where the blood showed this the egg-skin eschar was present; Dr. Watkins has repeatedly pointed out to me the syphilitic spore. Yet I must admit that the majority of my patients declared that there was no foundation for the suspicion of the disease. But when they received treatment for it, they were cured. Although some patients were honest in not knowing the history of their trouble, others did finally remember that they had contracted the disease, and others still acknowledged it at once.

So confident do I feel that my views are correct, that I now treat all cases of this kind with antisyphilitic remedies, and I find that a large percentage of them are benefited. In several cases I have been misled, and diagnosed suppurative gingivitis as pyorrhœa alveolaris. This I did because I could not find the egg-skin eschar, and when the blood was examined and seemed to substantiate my suspicions I refrained from giving specific treatment. To settle the question, I placed several patients suffering from suppurative gingivitis under specific treatment. This caused such unfavorable symptoms that I was soon forced to abandon it. In one case where the alveolar process, on the palatal surface of the teeth, was nearly destroyed and where it was practically in a normal condition on the buccal and labial surfaces, I was puzzled to know why this affection was not general. When septic pulps, salivary calculi, and syphilis were excluded, I concluded the trouble to be caused by the pressure from a vulcanite plate to which was attached an artificial velum that had been worn twenty years.

In another case, the cast of which I have here, the disease was extreme in character. There was great destruction of the interdental process, accompanied with a discharge of pus. Many of the teeth could readily be forced by the finger one-eighth of an inch farther into the socket. A tumor, osseous in character, extended along nearly the entire length of the alveolar process, on the buccal and labial surfaces, of the upper alveolar ridge. There was, however, a break in the line of the tumor between the right central and right lateral incisor. The right central incisor had been extracted several years earlier. In this space was an artificial crown attached to a small bridge-piece, as indicated on this cast.

The cast of the lower jaw showed, by the hypertrophied condition of the gum, the extent of the pocket. At first this patient persistently denied ever having syphilis, but the evidence of it was proved by examination of the blood. After I had gained the confidence of the patient, however, he admitted that he had contracted the disease a dozen years before, but had been under treatment for it. He gave as a reason for denying the fact that he did not wish it known to any one except his physician, who had positively stated that he was absolutely cured. The patient now returned to this physician, told him my views as to the cause of the tumefaction, and that I said he was still suffering from the taint. The physician made light of the diagnosis, and persuaded the patient not to return to me.

I regret not having an opportunity to finish the treatment of this case, as it would have been an excellent support to my belief that this class of tumors is the result of this dreadful poison.

It is fair to state, however, that within a year the health of the patient so completely failed that he was advised to visit the Hot Springs for syphilitic treatment.

When rheumatism is found to be present in a large percentage of cases, I believe it to be a coincidence, though not the cause. I believe that syphilis so reduces the resisting power of the constitution that rheumatism more easily steps in, much the same way that it may while the system is under any degenerating influence. I do not wish it understood that I believe pyorrhœa alveolaris exists in every case of syphilis, nor that syphilis is found in every case of pyorrhœa. But what I do believe is that some form of syphilis may exist in nearly all obstinate cases of pyorrhœa alveolaris that cannot otherwise be proved. As proof of this condition I mention such cases as do get well and remain so when placed under specific treatment until all signs of syphilis cease to appear, not only outwardly, but when the blood fails to show any evidence of it whatever. The value of blood examination, which tells when to commence treatment and when to cease treatment in this, as in some other diseases, is evident. I also regard it to be of great importance in diagnosing remote causes. Indeed, I will predict that the time is not far off when examination of the fresh specimen of the blood will be the principal evidence in proper diagnosis. I have sometimes thought that locolosis or pyorrhœa alveolaris may be caused by mercurial poison; but investigation does not bear out this surmise, for I have found this disease where there has been no history of mercury given.

Is it not, therefore, reasonable to conclude that this form of this disease is aggravated, if not caused by tertiary syphilis?

